# Enhanced Resistance to Leaf Fall Disease Caused by *Phytophthora palmivora* in Rubber Tree Seedling by *Sargassum polycystum* Extract

**DOI:** 10.3390/plants8060168

**Published:** 2019-06-11

**Authors:** Khemmikar Khompatara, Sittiporn Pettongkhao, Arnannit Kuyyogsuy, Nuramalee Deenamo, Nunta Churngchow

**Affiliations:** 1 Office of Agricultural Research and Development Region 8, Department of Agriculture, Ministry of Agriculture and Cooperatives, Hat-Yai, Songkhla 90110, Thailand; kjoy2000@hotmail.com; 2 Department of Biochemistry, Faculty of Science, Prince of Songkla University, Hat Yai, Songkhla 90112, Thailand; sit_ti_pon@windowslive.com; 3 Department of Chemistry, Faculty of Science and Technology, Nakhon Si Thammarat Rajabhat University, Nakhon Si Thammarat 80280, Thailand; arnannit.k@gmail.com; 4 Faculty of Science and Technology, Princess of Naradhiwas University, Narathiwat 96000, Thailand; nuramalee.dee@gmail.com

**Keywords:** biocontrol agent, defense response, induced resistance, *Hevea brasiliensis*, seaweed extract

## Abstract

The brown seaweed (*Sargassum polycystum* C. Agardh-Sargassaceae) extract was examined for its bioelicitor properties in the rubber tree seedling (*Hevea brasiliensis* (Willd. ex A.Juss.) Müll.Arg. - Euphorbiaceae) and its application to reduce the leaf fall disease caused by *Phytophthora palmivora* (Edwin John) Butler, 1917 (Peronosporaceae). The major purpose of this study was to apply this seaweed extract (SWE) to improve the disease resistance in rubber tree seedling compared to a chemical fungicide (1% metalaxyl). After foliar spraying of SWE solution, two antioxidant enzymes, catalase (CAT) and peroxidase (POD) and systemic acquired resistance (SAR)-triggered enzyme, β-1,3-glucanase (GLU), were analyzed. Both secondary metabolites, a phytoalexin scopoletin (Scp) and a signaling molecule salicylic acid (SA) were measured by high performance liquid chromatography (HPLC). Both SWE- and metalaxyl-treated plants had a close disease index (DI)-score which were 16.90 ± 1.93 and 15.54 ± 1.25, respectively, while the positive control sprayed with *P. palmivora* showed DI-score of 29.27 ± 1.89 which was much higher than those treated with SWE or fungicide. CAT, POD, and GLU were increased in rubber tree leaves treated with SWE solution. Furthermore, Scp and SA were significantly increased in SWE-treated leaves. Enhanced systemic acquired resistance induction, 2.09 folds of SA accumulation, was observed in the distal area comparing to the local area of SWE application. In conclusion, the positive effects of SWE elicitation from these studies revealed that SWE could be used as an alternative biocontrol agent for foliar spraying to enhance the defense responses in rubber tree seedling against *P. palmivora*.

## 1. Introduction

The Para rubber tree (*Hevea brasiliensis* (Willd. ex A.Juss.) Müll.Arg.—Euphorbiaceae.) is an economic plant as it is an important source of natural latex. It is commonly cultivated in tropical and sub-tropical areas [1], including Thailand. During periods of prolonged wet weather and high humidity conditions persisting for several days facilitate one of the most frequent diseases which is abnormal leaf fall (ALF) caused by various *Phytophthora* species. The impacts of this disease on rubber production are not only on latex yield and growth reductions due to leaf fall [2] but also on the 5% to 10% increased cost of disease control for the farmers in Southeast Asia countries [3].

Nowadays, transgenic technology is progressing and can be used to produce disease resistance plants. However, the adoption of transgenic plants is still not accepted in many countries. So, one option to prevent disease is to encourage plant immunity without any genetic modification, such as induced resistance, might be more acceptable. Understanding the physiology and biochemistry of defense responses in plants may lead researchers to find an alternative way to protect the plant from its pathogen. 

The state of enhanced defensive capacity responding to appropriate stimuli established by plants is called induced resistance, which is a highly effective defense against a broad range of subsequent pathogen challenges [4]. Two forms of induced resistance are systemic acquired resistance (SAR) involving accumulations of pathogenesis-related (PR) proteins and salicylic acid (SA) [5] and induced systemic resistance (ISR) depending on jasmonic acid (JA) and ethylene (ET) pathways [6]. Although the identity of the long-distance signals responsible for SAR is unclear, it is accepted that SA is required to activate SAR in the remote tissue [7]. 

In terms of crop management, there are many kinds of substances used as disease controlling agents such as *Trichoderma* [8] and chitosan [9]. In particular, chemical fungicides are commonly used by farmers. The disadvantages of this approach are contamination in the environment, physicochemical property changes of the soils, and toxicity in other living organisms [10]. 

Biofactors or chemicals obtained from various sources that can induce physiological changes in targeted living organisms are called elicitors. Elicitors can induce morphological and physiological alterations and phytoalexin accumulation in plants. Biotic elicitors are derived from fungi, bacteria, viruses or herbivores, plant cell components, as well as chemicals released by plants at the attack site upon infection, whereas abiotic elicitors include metal ions and organic compounds [11]. Elicitors have been used as plant defense inducers because they are friendly stimulants for plants. 

The perception of elicitors induces many phenomena, including medium alkalinization, ion flux, cytoplasmic acidification, oxidative burst, and reactive oxygen species production [11]. After perception, there are many events which can be induced, such as SA and nitric oxide production, phytoalexin accumulation, JA and ET inductions [11]. Many marker plant defensive proteins, such as pathogenesis-related 1 (PR1), endo-1,3-β-D-glucanase (GLU), and thaumatin-like protein, are frequently used to illustrate a SA induction. In contrast, proteins involving with the JA signaling pathway are chitinase, hevein-like protein, and protease inhibitor [12]. In addition, defense-related enzymes, catalase which is a major H_2_O_2_ scavenger in plants [13] and peroxidase which plays important roles in many physiological processes, such as defense response against pathogen infection and lignification [14], are usually used for testing elicitor activity. Many elicitors induce plant defense responses in different ways [11], so it is important to understand the effect of the elicitor that is going to be used.

Cell wall and storage polysaccharides from many marine macroalgae including green, brown, and red seaweed, such as carrageenans, alginates, fucans, ulvans, and laminarin, have been considered as alternative sources of elicitors which can trigger plant defense responses against viral, fungal, and bacterial infections [15]. For rubber tree, sulfated polysaccharide, carrageenan from red seaweed, *Acanthophora spicifera* (Vahl) Børgesen, 1910 (Rhodomelaceae) has been reported to induce rubber tree defense responses against *Phytophthora palmivora* (Edwin John) Butler, 1917 (Peronosporaceae) infection [16]. 

The seaweed *Sargassum polycystum* C. Agardh-Sargassaceae is a largely unexploited brown seaweed in the *Phaeophyceae* class. It can be found wildly in enormous quantities almost all over the world. An important advantage of using the *Sargassum* biomass is that it contains many active compounds [17,18]. In previous studies, the sulfated fucan oligosaccharide of the other brown seaweed was found to induce several defense responses in many plant species [19,20]. 

The objective of this study was to elucidate the efficiency of seaweed extract (SWE) for controlling abnormal leaf fall disease caused by *P. palmivora* in rubber tree seedlings comparing to a commercial fungicide and also to determine the biochemical changes in the rubber tree seedlings, such as the activities of defense enzymes including catalase (CAT), peroxidase (POD), and β-1,3-glucanase (GLU), accumulations of secondary metabolites (scopoletin (Scp) and salicylic acid (SA)), and SAR induction following the foliar application of this SWE.

## 2. Results

### 2.1. Seaweed Extract and Chemical Composition

**Figure 1:**
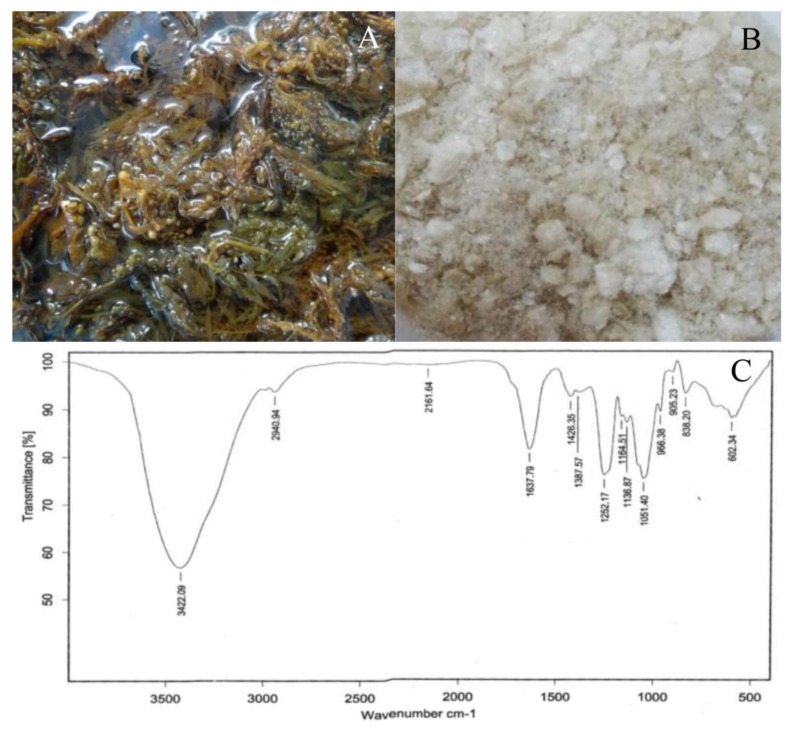
Fresh *Sargassum polycystum* C. Agardh (**A**), seaweed extract (SWE) of *S. polycystum* (**B**), and FTIR spectrum of SWE (**C**).

After extraction (Figure 1A,B), the quantity of SWE obtained was 3.10 ± 0.98 g from 100 g of dry seaweed (mean ± SD, n = 5). In the FTIR spectrum (Figure 1C), the presence of a broadly stretched peak around 3420 cm^−1^ and a small peak at 2940 cm^−1^ were due to the stretching vibrations of O–H and C–H, respectively [21]. The FTIR band around 1400 to 1470 cm^−1^ could be attributed to scissoring vibration of CH_2_ in galactose and mannose [22]. 

The absorption band at 1252 cm^-1^ was due to the presence of sulfated ester groups (S = O) (Figure 1C), which is a characteristic component in fucoidan [23]. Fucose generally has a strong absorbance at wave number of 1200 to 1050 cm^-1^ [8]. The absorption peak around 800 to 860 cm^-1^ may correspond to the S=O, which indicates the presence of esterified sulfate, generally observed in seaweeds [22]. The weak absorption band observed near 600 to 670 cm^-1^ was due to C-S and C=S stretchings [24]. Not only functional group detection but the chemical compounds in SWE were also determined as follows: total carbohydrates (45.83 ± 2.72 g/100 gdw), fucose (20.62 ± 0.33 g/100 gdw), sulfate (30.26 ± 0.86 g/100 gdw), uronic acid (12.17 ± 0.11 g/100 gdw), and total phenolic (68.95 ± 0.37 mg/100 gdw). These data were presented as mean ±SD from three individual analyses. 

### 2.2. Applications of SWE against P. palmivora Infection on Rubber Tree Seedlings

**Figure 2:**
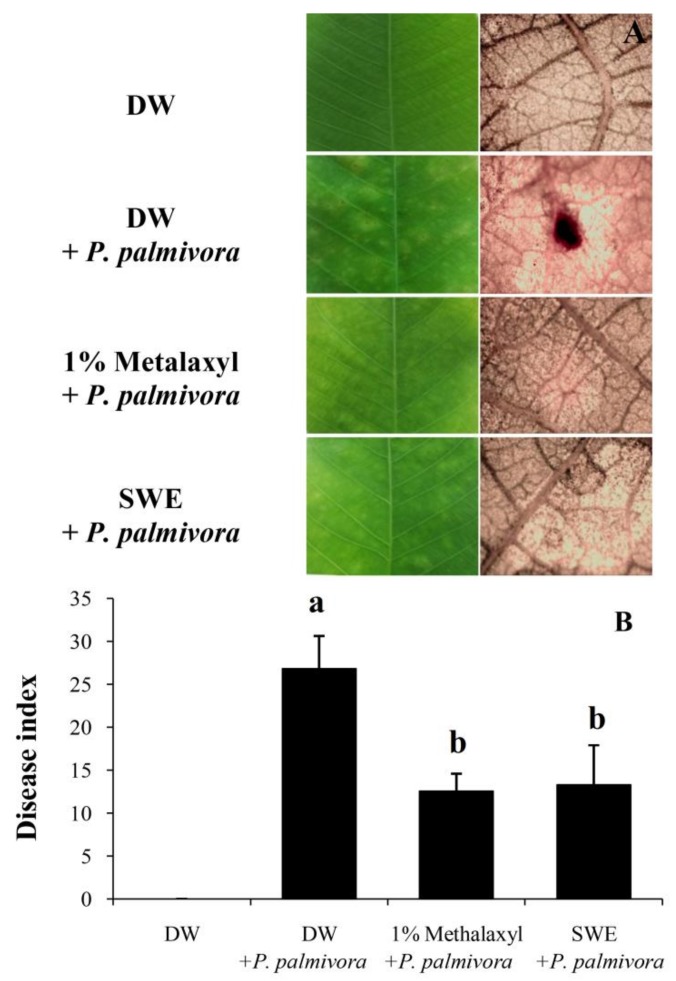
Leaf lesions and phloroglucinol stainings (**A**) and average disease index was calculated based on the percentage of leaf area effected using a 4 (0–3)-point disease rating scale (**B**) in four sets of elicitation bioassay at 5 DAI (n=5). According to Scheffe’s Multiple range test, different letters indicate significant differences among treatments (*p* > 0.05).

After treatment with SWE or fungicide (1% metalaxyl), the resistance to *P. palmivora* was investigated on both sets at 5 days after inoculation (DAI) compared to positive and negative control. The leaf lesion and phloroglucinol staining (Figure 2A) showed that only the positive control set was detected by the lignin staining solution. In particular, we performed three experiments, and all results exhibited homogeneity. 

**Figure 3:**
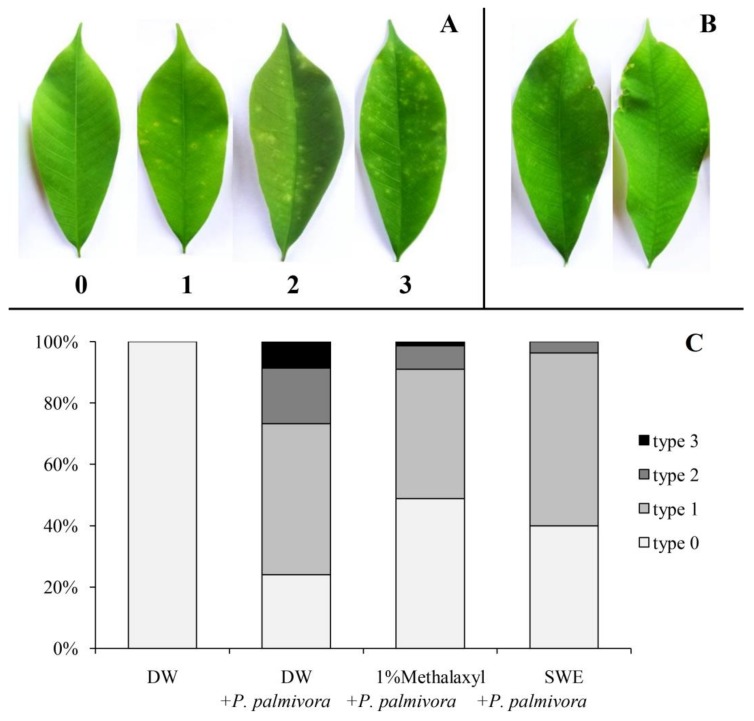
Disease development in the leaves at day 5 after inoculation could be divided into four types; 0 = no infection, 1 = light infection, 2 = moderate infection and 3 = severe infection (**A**); some damage leaves caused by 1% metalaxyl (**B**), and percentage of each type of leaves in each treatment (n = 120) (**C**).

For the infection test, the result showed that the SWE treatment gave the disease index score close to that of the metalaxyl treatment (16.90 ±1.93 and 15.54 ± 1.25, respectively). It was obviously lower than the positive control (29.27 ± 1.89), which was sprayed with *P. palmivora* (Figure 2B). For disease index score calculation, four types of infected leaves were classified as shown in Figure 3A, and the percentage of infected leaves in each type from whole leaflets of five experiments are shown in Figure 3C. The SWE and 1% metalaxyl seemed to reduce type 2 and 3 infected leaves, which were moderate and severe infections, respectively. Furthermore, the undesirable burning at the leaf margin was only found in leaves treated with 1% metalaxyl (Figure 3B). 

### 2.3. Induction of Defense-Related Enzyme Activity by SWE Comparing to P. palmivora

**Figure 4:**
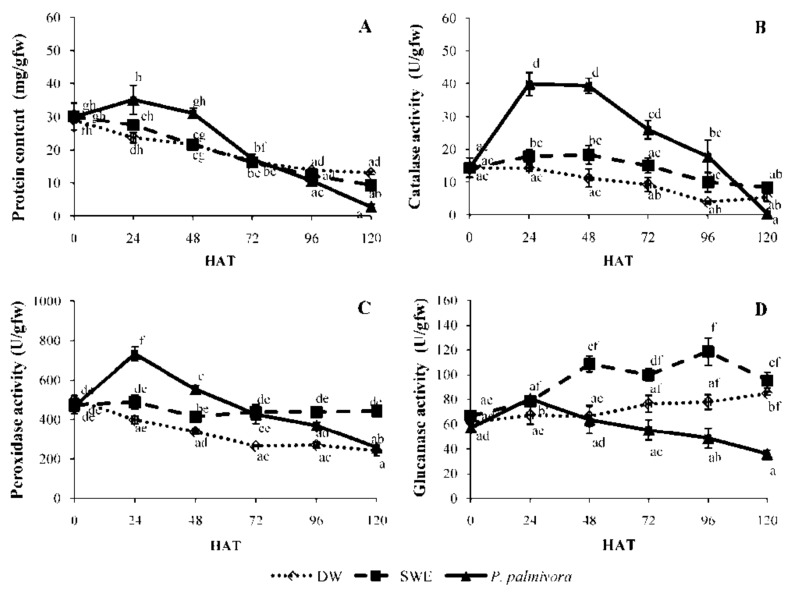
Protein content (**A**), catalase (CAT) activity (**B**), peroxidase (POD) activity (**C**), and β-1,3-glucanase (GLU) activity (**D**) in rubber tree leaves treated with 0.25 mg/mL SWE, 1x105 zoospores/mL *P. palmivora* and distilled water at 0, 24, 48, 72, 96 and 120 h after treatment (HAT). Data were presented as mean ± S.E. (n = 4). Statistical analysis was performed using a one-way ANOVA, taking *p* < 0.05. The letters indicate the significant differences among time points.

After spraying with the pathogen, SWE, or DW, the protein content in leaves treated with *P. palmivora* showed a significant increase within 24 h after treatment (HAT) then started to decline at 48 HAT, while the SWE-treated leaves and DW-treated leaves showed the same decreasing pattern. After 72 HAT, the level of protein in *P. palmivora*-treated leaves was rapidly decreased more than the SWE- and DW-treated leaves (Figure 4A).

CAT activity in *P. palmivora*-treated leaves showed a strong induction (39.90 ± 3.53 U/gfw) at 24 HAT and (39.36 ± 2.30 U/gfw) at 48 HAT then declined. In SWE-treated leaves, the CAT activity was slightly increased (17.92 ± 1.95 U/gfw) compared to the control (14.29 ± 1.08 U/gfw). For the control set, the increase in CAT activity was not found at any time point (Figure 4B).

POD activity in three sets of treatments (Figure 4C) revealed a different pattern. In pathogen-treated leaves, a significant increase of POD activity was shown by a single peak (731.76 ± 35.58 U/gfw) at 24 HAT and decreased after that, while the POD activity in SWE-treated ones did not change significantly (approximately 448.81 ± 11.71 U/gfw) throughout the study period. In the control set, the POD activity showed slowly decreases from 0 to 120 HAT (Figure 4C).

The GLU activity (Figure 4D) in the *P. palmivora*-treated leaves reached the maximum level (80.49 ± 1.89 U/gfw) within 24 HAT then sharply decreased. In SWE-treated set, GLU activity was strongly enhanced (108.63 ± 6.4 U/gfw) at 48 HAT and reached the highest level (118.65 ± 11.22 U/gfw) at 96 HAT, while the DW-treated set showed slowly increase of GLU activity from 0 to 120 HAT. 

### 2.4. Enhancement of the Secondary Metabolite Accumulation by SWE

**Figure 5:**
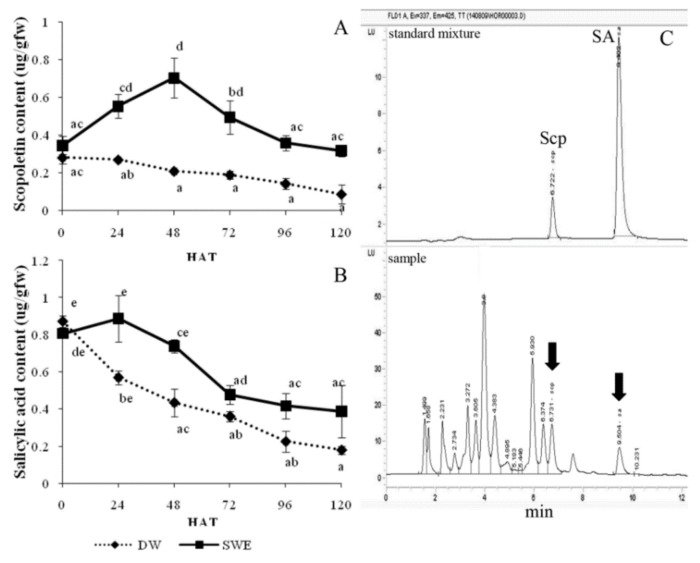
Scopoletin (Scp) content (**A**) and salicylic acid (SA) content (**B**) in rubber tree leaves after being treated with 0.25 mg/mL SWE and distilled water at 0, 24, 48, 72, 96 and 120 h after treatment (HAT). HPLC Chromatograms obtained from a Scp and SA standard mixture and leaves sample extract (**C**). Data were presented as mean ± S.E. (n = 3). Statistical analysis was performed using a one-way ANOVA, taking *p* < 0.05. The letters indicate the significant differences among time points.

The enhancement of Scp accumulation by SWE is shown in Figure 5A. The result indicated that the SWE could induce the Scp accumulation in the leaves to the maximum level (0.70 ± 0.11 µg/gfw) at 48 HAT, while this metabolite in the control leaves was decreased gradually from 0 to 120 HAT (Figure 5A). 

For SA accumulation determination, the SA content was slightly increased (0.89 ± 0.12 µg/gfw) at 24 HAT and declined after that, while the control was gradually reduced from 0 to 120 HAT. However, the amount of SA in the SWE-treated leaves was higher than that of the control at all-time points (Figure 5B).

### 2.5. Induction of Systemic Acquired Resistant (SAR) by SWE

**Figure 6:**
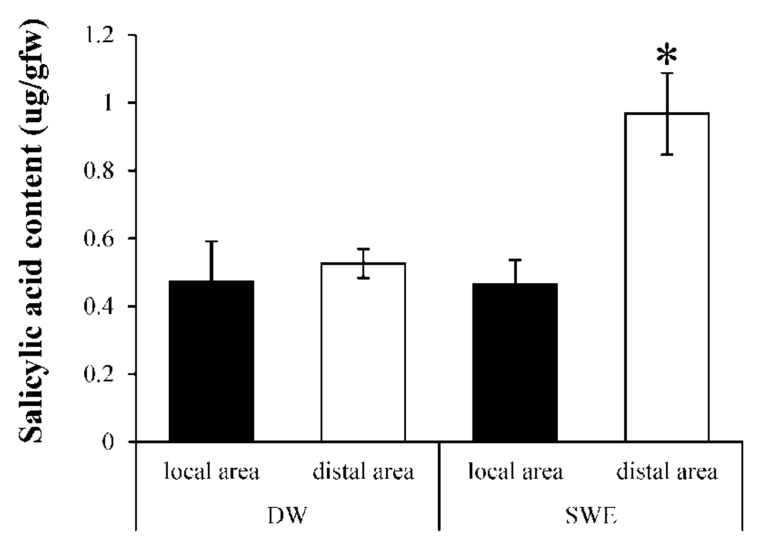
Salicylic acid (SA) contents in SWE-treated leaves. The black bars refer to SA content in the lower leaves (local area) sprayed with SWE or distilled water for 24 h, and the white bars refer to SA content in untreated upper leaves (distal area) of each set. Data were presented as mean ± S.E. (n = 3). Statistical analysis was performed using a one-way ANOVA, taking *p* < 0.05.

To evaluate the induction of SAR by SWE, the amount of SA was measured between two distinct areas. The result showed that SA content in the SWE-treated local area was 0.46 ± 0.07 µg/gfw which was lower than that in the distal area (0.97 ± 0.12 µg/gfw) (Figure 6). The induction effect was found only in the SWE-treated set but not found in the control set. 

## 3. Discussion

Both FTIR and colorimetric analyses of SWE indicated the presence of carbohydrate, fucose, galactose, sulfate, uronic acid, and phenolics which was similar to those reported in other studies [18,22] although the concentration is different which depends on the habitat, harvesting season, and extraction methods. 

The application of this SWE was evaluated by the process of induced resistance in rubber tree seedlings in comparison to the commercial fungicide (1% metalaxyl). The result suggested that both SWE and 1% metalaxyl could reduce disease severity by about 50% compared to the positive control; in addition, no lignin was detected in these protected leaves (Figure 2A,B). It has been shown that many types of seaweed induce plant defense responses against pathogen infection. For example, glucuronan and ulvans from the green alga, *Ulva lactuca*, induce tomato seedlings resistance to *Fusarium oxysporum* f. sp*. Lycopersici* infection [25]. Carrageenans, alginates, fucans, ulvans, and laminarin from many marine macroalgae, including green, brown, and red seaweed can trigger plant defense responses against viral, fungal, and bacterial infections [15]. For rubber tree, sulfated polysaccharide, carrageenan from red seaweed, *A. spicifera* has been reported to induce rubber tree defense responses against *P. palmivora* infection [16].

The protein content in pathogen-treated leaves was significantly increased within 24 HAT, while the SWE-treated and control leaves were decreased. This result might be described in terms of energy balance between growth and defense support within the plant. After pathogen attack, the activation of plant defense responses places a high metabolic demand; for example, the production of PR proteins may reach to 10% of the total soluble proteins of an infected leaf [26]. On the other hand, under pathogen-free conditions, the activation by elicitor does not damage the plant tissues, thereby alleviating the synthesis of defense compounds. 

The perception of elicitors induces many phenomena, including medium alkalinization, ion flux, cytoplasmic acidification, oxidative burst, and reactive oxygen species production [11]. CAT is a major H_2_O_2_ scavenger in plants [13]. It is normally involved in the elimination of H_2_O_2,_ which is routinely generated at a low level. During a pathogen attack, the H_2_O_2_ and another member of reactive oxygen species (ROS) are synthesized at the wound site to restrict the spread of infection [27]. In this study, the CAT activity in *P. palmivora*-treated leaves rapidly increased within 24 HAT (Figure 4B). Therefore, the ROS produced in this period might activate the raising of CAT activity. However, the CAT activity in SWE-treated leaves showed a slight increase. This result suggested that H_2_O_2_ might be produced at the low level in this condition.

POD is known as pathogenesis-related protein 9 (PR9). This enzyme seems to be involved in the control of ROS levels generated by wound-affected cells in antioxidant systems contributing to the early stress response, signaling, healing, or cell death process [28]. In this study, the pathogen treatment showed the rapid induction of POD activity, reaching the maximum level within 24 HAT (Figure 4C). This result could be described by the healing process that required higher activity of POD, and it may be raised for controlling the ROS in the antioxidant system. Peroxidase also plays important roles in various physiological processes, including lignification and defense response against pathogen infection [14]. In our results, the accumulation of lignin in rubber leaves treated with *P. palmivora* (Figure 2A) could be the results of POD induction (Figure 4C). However, the POD activity pattern in the SWE-treated leaves could infer to the low level of ROS generated after elicitation. The POD activity in SWE-treated leaves was not much higher than that in DW-treated leaves but kept synthesizing throughout 120 HAT (Figure 4C). Similarly, the SPS sulfated polysaccharide from *A. spicifera* also reported as POD inducer in rubber tree [16]. 

The induction of glucanase (GLU) which is known as pathogenesis-related protein 2 (PR2) has been reported during a variety of pathogen infections in many plants, such as apple [29], tomato [30], and rubber tree [31]. In our study, the GLU activity was increased within 24 HAT (Figure 4D). In particular, the GLU activity in SWE-treated leaves was exhibited at higher level (~ 2 folds) and more prolong (48–96 HAT) than leaves treated with *P. palmivora*. The GLU activity which was increased in the leaves treated with SWE are also the member of SA-triggered SAR, and it could inhibit the growth of *P. palmivora* by hydrolyzing the β-1,3-glucan which is a major structural component belonging to the class Oomycetes [32]. Rubber tree leaves treated with sulfated polysaccharide from *A. spicifera* induced GLU expression and resisted to *P. palmivora* infection [16]. 

According to the secondary metabolite analyzed by HPLC, the increase of two compounds was detected after SWE elicitation. The first metabolite compound was Scp which has antimicrobial properties and antioxidant capacity by scavenging the ROS in plants [33]. In this study, after treatment with SWE, high Scp accumulation appeared within 48 HAT (Figure 5A). It has been shown that rubber tree leaves treated with red seaweed extract from *A. spicifera* caused Scp accumulation [16]. In addition, Scp was strongly enhanced after rubber tree leaves were infected with *P. palmivora* or rubber tree cell suspension was treated with a protein elicitor extracted from *P. palmivora* in our previous experiments [34,35]. It has been shown that Scp can be synthesized upon a plant response to biotic or abiotic stress [33]. The second metabolite compound was SA, which was involved in the SAR induction [5,36]. The SA content was slightly increased at 24 HAT and higher than in DW-treated leaves throughout 120 HAT (Figure 5B), indicating that SWE could induce SA in rubber tree leaves. Since PR1, GLU, and thaumatin-like protein are frequently induced by the SA signaling pathway [12], so the induction of GLU activity in our study (Figure 4D) might be triggered by SA which was raised after SWE treatment (Figure 5B). 

The effect of SWE on the resistance may act through the accumulations of secondary metabolite Scp, which could inhibit *P. palmivora* directly and SA, which triggers the SAR and leads to the accumulation of many defense proteins. Plants can produce Scp, a coumarin phytoalexin, which plays as an antimicrobial substance triggered by pathogen infection or elicited by abiotic agents. In addition, the increasing activity of POD and CAT after SWE induction might support the elimination of the ROS after pathogen attack. 

Systemic acquired resistance (SAR) involves an accumulation of PR proteins and SA [5], whereas induced systemic resistance (ISR) depends on JA and ET pathways [6]. Even though the identity of the long-distance signals responsible for SAR is unclear, it is accepted that SA is required to activate SAR in the remote tissue [7]. We measured the SA levels accumulated in local and distal leaves after pretreatment with SWE and DW. In this study, the SA content was present in the distal area of leaves treated with SWE about 2 folds, which was higher than that observed in the local area (Figure 6). This result suggested that SWE could induce SAR in rubber tree leaves. It has been reported that sulfated fucan oligosaccharides induce SA accumulation at a systemic level and enhanced protection against tobacco mosaic virus (TMV) infection [37].

## 4. Materials and Methods

### 4.1. Seaweed Extraction

The brown seaweed *S. polycystum* was obtained from the south-east coast of Thailand. The extraction method was modified from Rioux et al. [38]. Briefly, the oven-dried material was mixed with 1% (w/v) CaCl_2_ at 85 °C for 4 h, and then centrifuged at 10,000 rpm for 30 min. The supernatant was filtrated through Whatman No.4 filters before mixing with 1 volume of 2% NaCl and 2 volumes of 95% EtOH and stirred for 1 h at room temperature, then stored at −20 °C for 48 h. The mixture was centrifuged at 10,000 rpm for 30 min, and the supernatant was collected. EtOH was evaporated from the supernatant then the resulting supernatant was dialyzed with 2 kDa MW cut-off membrane and freeze-dried. Finally, the dried seaweed extract was dissolved in distilled water to prepare the SWE solution stock, autoclaved at 121 °C for 20 min to eliminate microorganisms and stored at −20 °C until use. 

### 4.2. Fourier Transform Infrared (FTIR) Analysis

The SWE was ground with potassium bromide pellet and characterized by using Fourier Transform Infrared Spectrometer, VERTEX 70, Bruker, Germany between 400 and 4000 cm^−1^. The measurements were done at 32 scans/sample and a resolution of 4 cm^−1^.

### 4.3. Chemical Composition Determination

The SWE was determined for its carbohydrate content by the phenol-sulfuric acid procedure [39]. The fucose content determination was adapted from the Dische method [40], and the amount of fucose was calculated from a standard curve of L-fucose. Sulfate in the SWE was based on the barium sulfate (BaSO_4_) determination using barium chloride (BaCl_2_) [41]. The uronic acid content was analyzed using a carbazole method modified from Bitter et al. [42], and the uronic acid content was calculated comparing to a standard curve obtained from D-glucuronic acid solutions. Phenolic content was measured according to the method described by Torres et al. [43], and results were presented in term of milligram gallic acid equivalent per 100 g dry weight of extract (mg GAE/100 gdw). 

### 4.4. Biological Property Determination

#### 4.4.1. Pathogen and Zoospore Preparation

For zoospore preparation, the mycelium of *P. palmivora* grown on a potato dextrose agar (PDA) was transferred to a V8 agar and incubated at room temperature for 1 week. After that, 10 mL of sterile distilled water was added to promote sporangial growth for 2 days. The mobile zoospores were liberated by using cold treatment. A number of released zoospores were observed and counted using hemocytometer under the microscope (10×).

#### 4.4.2. Plant Treatment

The healthy bud-grafted seedlings of *H. brasiliensis* susceptible cultivar RRIM 600 were propagated in soil and raised in polyethylene bags in the nursery for 2 weeks. The homogeneous seedlings were selected and transferred to the condition-controlled room which was maintained at 25 to 28 °C with 12 h fluorescence light for 1 day before treatment. For each experiment, the volume (10 mL) of 0.25 mg/mL SWE, 1 × 105 zoospores/mL of *P. palmivora*, 1% metalaxyl (commercial fungicide) or distilled water was fixed for each seedling treatment.

For protein content and enzymatic activity detections, all seedlings were divided into 3 sets, then the first set was sprayed with SWE solution, the next set was sprayed with *P. palmivora* solution, and the remains were sprayed with distilled water as a negative control set. Leaflets from 15 seedlings per treatment were taken at 0, 24, 48, 72, 96, and 120 h after treatment (HAT). After weighting, all samples were frozen in liquid N_2_ and stored at −20 °C before use. Each value in this experiment was derived from 4 replicates.

The secondary metabolite accumulations induced by SWE were also investigated. For this study, the seedlings were sprayed with SWE solution or distilled water. The leaf samples from both treatments (15 seedlings/treatment) were taken at different time intervals (0, 24, 48, 72, 96, and 120 HAT) for Scp and SA determinations. Data were obtained from 3 independent replicates.

For SAR induction determination, SWE solution was sprayed onto the lower leaves of seedlings (position 4 from top leaves and all lower leaves), while the upper leaves (position 1 to 3 from top leaves) were covered with a plastic bag to prevent the SWE solution or the distilled water which was used for the control set. Six hours after spraying, the plastic bags were removed. At 24 HAT, SA contents in the local area and the distal area were extracted and measured by HPLC. Each value in this experiment was derived from 3 replicates, and each replicate contained 4 seedlings.

#### 4.4.3. Extraction of Defense-Related Enzyme Activity 

Sample extraction was performed according to the procedure of Chanwun et al. [44] with some modifications. In detail, 0.5 g of leaves were ground in liquid N_2_ and blended with 30 mg of polyvinylpolypyrrolidone (PVPP) using a mortar and pestle. The powders were mixed with 1 mL of a 0.1 M Tris–HCl buffer, pH 7.0, containing 0.25% (v/v) Triton X-100. After centrifugation (12,000 rpm at 4 °C for 30 min), the supernatant was used as a crude extract for enzyme activity assay. Only for GLU activity assay, the protein in the crude extract was subsequently precipitated in 90% (w/v) ammonium sulfate then the resulting pellet was suspended in 0.1 M Tris–HCl buffer, pH 7.0 and dialyzed through cellulose membrane tubing with a 12 kDa MW cut-off for 24 h.

#### 4.4.4. Protein Content 

The protein content in extracted samples was determined using spectrophotometry at 595 nm, according to the method of Bradford [45] using bovine serum albumin (BSA) as standard.

#### 4.4.5. Enzyme Assays 

The activity of catalase (CAT) was determined as described by Brennan and Frenkel [46] with some modifications. The reaction mixture containing 950 µL of 1 mM H_2_O_2_ in 50 mM Tris–HCl pH 6.8 and 50 µL of the diluted enzyme was incubated for 10 min at room temperature. After that, 25 µL of 20% (v/v) TiCl_4_ in concentrated HCl was added to stop the reaction. The remaining H_2_O_2_ from the reaction was measured at the absorbance at 415 nm. One unit of CAT activity equaled to one micromole of H_2_O_2_ that was lost per min. The result was reported in term of total activity (U/gfw).

The activity of peroxidase (POD) was assayed as described by Shannon et al. [47] with some modifications. The reaction mixture contained 2.75 mL of 0.05 M sodium acetate buffer pH 5.4, 100 μl of 0.25% (w/v) o-dianisidine (ε = 11.3/mM/cm at 460 nm), 100 μl of 0.1 M H_2_O_2_, and 25 μl of enzyme solution. The changes in absorbance at 460 nm was recorded every 15 s for 1 min. The result was reported in term of total activity (U/gfw). 

The glucanase (GLU) activity was assayed based on the colorimetric method describes by Santos et al. [48] with some modifications. The reaction consisted of 100 µL dialysate, 200 µL of 0.1 M sodium acetate buffer pH 5.0, and 200 µL of 2 mg/mL laminarin and then incubated at 35 °C for 15 min. The reaction was stopped by adding 200 μl of dinitrosalicylate and boiled for 5 min. After that, the reaction solution was diluted with 200 µL of distilled water, and the absorbance was measured at 540 nm. For activity calculation, two blanks were prepared, one had no sample in the reaction, and the other had no laminarin in the reaction. An activity unit was defined as the amount of enzyme that produced reducing sugars equivalent to 1 µg of glucose per min under assay conditions. The result was reported in term of total activity (U/gfw).

#### 4.4.6. Scopoletin (Scp) and Salicylic Acid (SA) Contents in Leaves

Scp and SA were extracted from 0.5 g leaves according to the modified method of Ederli et al. [49]. After blending the leaves in liquid N_2_, 750 µL of 90% MeOH was added to the mortar. The homogenate was transferred to a 1.5 mL Eppendorf tube and centrifuged at 12,000 rpm for 5 min. The supernatant was collected, while the pellet was mixed with 500 µL of 100% MeOH and centrifuged at 12,000 rpm for 5 min. All supernatant fractions were pooled and 50% (w/v) trichloroacetic acid was added to adjust the final concentration of 5% (w/v). All samples were filtered through 0.2 µm nylon filters before injection. 

The amount of Scp and SA were determined by high-performance liquid chromatography (Agilent 1100) using a reverse phase column (ZORBAX Eclipse XDB-C18, 4.6 × 150 mm, 5 microns). The acetonitrile (ACN) and 0.1% formic acid were used as a mobile phase. The gradient program was set as follows (time in min/percentage ACN): 0–2/80, 8.5–10/60, 12/55, 13/40, and 15/15 with 1 mL/min flow rate at 40 °C (Figure 3C). The fluorescence detector was set for Scp using Ex = 337 nm and Em = 425 nm and for SA using Ex = 294 nm and Em = 426 nm. All data were analyzed using Chemstation Software (Agilent Technologies).

### 4.5. Applications of SWE against P. palmivora Infection on Rubber Tree Seedlings

The healthy bud-grafted homogeneous seedlings with 2 week-old leaves from the nursery were used for this experiment, which consisted of 4 treatments. Each treatment consisted of 5 replicates, and twenty-four leaflets from each replicate were used to calculate for the DI scores. The first and second treatment was sprayed with distilled water for negative and positive control, respectively. The third treatment was sprayed with SWE solution, and the last treatment was sprayed with 1% (w/v) metalaxyl. After 24 h of elicitation, all treatments (except negative control set) were inoculated with *P. palmivora* solution (1 × 105 zoospores/mL), Then covered with a clear plastic box to maintain the required humidity. The disease severity was evaluated at 5 days after inoculation in term of disease index (DI) score based on Parry [50] (a scale 0 = no infection, 1 = light infection, 2 = moderate infection, and 3 = severe infection). The disease index (DI) was calculated (sum of disease ratings of individual leaves/total number of leaves) × (100/maximum disease category). 

### 4.6. Lignin Staining

Lignin staining on the leaves was observed using phloroglucinol-HCL staining method [51]. The leaf pieces were placed on the petri dish containing staining solution for 2 to 3 min. After that, the leaf piece was observed under the microscope (10×). Lignin deposition was visualized as the red color.

### 4.7. Statistics

All data were tested for homogeneity and analysis using analysis of variance (ANOVA). Multiple comparison (Scheffe) tests were performed when significant (*p* < 0.05) differences between means were detected by ANOVA.

## 5. Conclusions

In conclusion, this SWE could enhance rubber tree against *P. palmivora* infection. It slightly induced CAT and POD and highly induced GLU. Scp and SA accumulations were also triggered by SWE, and the SA content was obviously detected in the distal area. Understanding these elicitor effects and the way to manipulate them would render an important tool for serving cost-effective and eco-friendly disease management, supporting the organic farming policy in many countries, and approaching sustainable agriculture. In future, the aim to use SWE as the alternative biocontrol agent will be needed to verify the effect on plant disease control under field conditions or may be applied to a variety of crops protection.
